# Neonatal and Pediatric Emergency Room Visits in a Tertiary Center during the COVID-19 Pandemic in Italy

**DOI:** 10.3390/pediatric13020023

**Published:** 2021-04-07

**Authors:** Davide Silvagni, Laura Baggio, Patrizia Lo Tartaro Meragliotta, Pietro Soloni, Giovanna La Fauci, Chiara Bovo, Stefania Ielo, Paolo Biban

**Affiliations:** 1 Paediatric Emergency Room, Department of Neonatal and Paediatric Critical Care, University Hospital of Verona, Piazzale Stefani 1, 37126 Verona, Italy; davide.silvagni@aovr.veneto.it (D.S.); laura.baggio@aovr.veneto.it (L.B.); patrizia.lotartaromeragliotta@aovr.veneto.it (P.L.T.M.); pietro.soloni@aovr.veneto.it (P.S.); giovanna.lafauci@aovr.veneto.it (G.L.F.); ielo.stefania42@gmail.com (S.I.); 2 Medical Direction, University Hospital of Verona, Piazzale Stefani 1, 37126 Verona, Italy; mcq142002@yahoo.it

**Keywords:** newborn, child, pediatric emergency department, triage code, COVID-19

## Abstract

The COVID-19 pandemic is affecting healthcare services worldwide. We investigated the impact of a strict lockdown policy on the characteristics of neonatal and pediatric attendances to our pediatric emergency department (PED). The clinical features of PED visits in March–April 2020 (COVID-19) and March–April 2019 (non-COVID-19) were analyzed. During the COVID-19 lockdown period, visits reduced by 67%, from 3159 to 1039. Neonatal access decreased from 78 to 59, mainly due to fewer pathological conditions, with a complete disappearance of respiratory infections. On the other hand, minor neonatal clinical conditions rose from 44 (56.4%) to 48 (81.4%), mostly due to feeding-related issues. Communicable diseases, particularly respiratory infections and gastroenteritis, dropped from 1552 (49.1%) to 288 (27.7%). Accident-related visits also decreased during COVID-19, from 535 (16.9%) to 309 (29.7%), becoming the most common cause of PED access. Hospital admissions reduced from 266 to 109, while PICU (pediatric intensive care unit) admissions decreased from 27 to 11, with a comparable rate of 10.1% in both periods. The lockdown due to COVID-19 had a substantial impact on our PED visits, which markedly decreased, mainly due to fewer respiratory infections. Unexpectedly, neonatal visits for minor conditions did not decline, but rather slightly increased. Among the children admitted to the PICU, none had respiratory disease.

## 1. Introduction

On 30 January 2020, the World Health Organization (WHO) declared the outbreak of the novel coronavirus SARS-CoV-2 as a “public health emergency of international concern”, and on 11th March, as a global pandemic [1]. Italy was the first country to report cases of COVID-19 in Europe, starting in the northern regions [2,3]. From 23rd February, a shelter-in-place order, including restriction of all social and non-necessary activities, school closures, and limitation in public transport, was imposed in outbreak areas to reduce the spread of SARS-CoV-2 and other viral infections mimicking COVID-19. From 1st March, these progressively restrictive measures for social contacts were extended all over the country, including a national lockdown, established on 9th March and strictly maintained until 27th April. From the first days of the COVID-19 emergency, due to the high risk of contagion, visits to hospitals and emergency departments (ED) were discouraged, except for urgent reasons. As a consequence, several pediatric emergency departments (PED) experienced a marked reduction in visits during the pandemic (−73% and −88% compared to same periods in 2019 and 2018, respectively) [4,5]. Furthermore, cases of delayed access to PED were reported, including among severely ill children [4]. At the same time, medical assistance by primary care physicians was abruptly limited only to phone consultations, especially for children with any suspected infectious disease.

The aim of this study was to analyze the variation in terms of volume, characteristics, and triage code severity of neonatal and pediatric access to our pediatric emergency department (PED), in relation to the strict lockdown policy in a COVID-19 outbreak area in northern Italy. To this end, we compared epidemiological and clinical data observed during the same period in the previous year, to investigate possible causes of discrepancies and identify potential interventions to optimize the healthcare system’s response, should another lockdown occur in the future.

## 2. Materials and Methods

### 2.1. Study Design

We performed a retrospective observational study in the PED of the Verona University Hospital during the strict COVID-19 lockdown period, starting in February 2020 and ending in April 2020. Our PED is a tertiary center, providing care to about 20,000 children per year, for any pathological conditions but major trauma. In our center, according to patients’ symptoms and vital signs, the triage code severity is defined by color codes as follows: white for non-urgent, green for deferrable urgency, yellow for urgent, and red for emergency.

Following any PED access, patients may be discharged home or admitted, either to pediatric wards or to the pediatric intensive care unit (PICU).

We analyzed the data of all patients who accessed our PED from 1 March to 30 April 2019 (non-COVID-19 period), and from 1 March to 30 April 2020 (COVID-19 period), when stringent restrictive policies for social distancing and other mitigation procedures had been established nationwide.

We also retrieved the number of PED visits in a two-month span (January and February) in 2019 and 2020 to compare the trend of PED accesses before the COVID-19 season.

Data were retrieved from our electronic PED database, including details on the number of visits, age and sex distribution, urgency at triage, discharge diagnosis, and outcome.

According to age, patients were split into seven subgroups, from below 28 days of life up to more than 14 years (Table 1). Newborns (<28 days of life) were subdivided in two categories: infants with pathological conditions or infants with minor clinical issues (Table 2).

Discharge diagnoses were subdivided into eight categories: (1) communicable diseases (respiratory and gastrointestinal infections, aspecific fever); (2) other body system diseases (gastrointestinal, genitourinary and cutaneous); (3) seizures, epilepsy and neurological conditions; (4) headache; (5) accidents (head injury, other traumas-burns-wounds, foreign body ingestion-inhalation, musculoskeletal pain); (6) psychiatric disorders; (7) acute surgical conditions; and (8) other conditions (Table 3).

The outcome of patients was classified into three categories: discharged home, admission to a pediatric ward, or admission to the PICU.

We compared all data related to the non-COVID-19 period (2019) and the COVID-19 period (2020).

### 2.2. Statistical Analysis

Descriptive statistics were reported as the median and range or mean and standard deviation for continuous variables, and proportion and percentage for categorical variables. The Mann–Whitney U test was used for group comparison analysis of continuous variables, and the chi-squared and Fisher’s exact tests for categorical variables. When the *p*-value was < 0.05, the difference was regarded as statistically significant. All statistical tests were 2-tailed. All statistical analyses were performed using Stata Version 13.0 (StataCorp, College Station, TX, USA).

## 3. Results

### 3.1. Characteristics of PED Visits in the Two Study Periods

A total of 4198 visits were registered in the two study periods. PED visits markedly decreased from 3159 in 2019 (1605 in March, 1554 in April) to 1039 in the COVID-19 period (524 in March, 515 in April) (Table 1).

**Figure 1:**
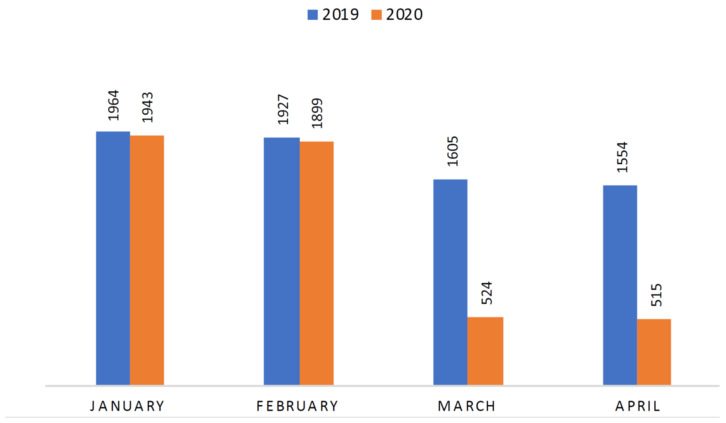
Monthly Pediatric Emergency Department visits from January to April in 2019 and 2020, respectively.

PED visits registered in January–February 2019 were similar to those observed in the same period in 2020 (3.891 vs. 3.842, respectively) (Figure 1).

The sex distribution was comparable, with a predominance of males in both epochs.

On average, the number of PED visits per day was significantly higher in the non-COVID-19 period (51.6, +10.4) compared to the COVID-19 period (17 + 6.8) (*p* < 0.001). At triage, white and green codes significantly decreased in the COVID-19 period. Yellow codes dropped from 261 to 100, while a single red code was observed in each epoch (Table 1).

### 3.2. Distribution of Visits According to Age Group and Disease Category

In the COVID-19 period, the number of visits decreased in all age groups, albeit unevenly. For the neonatal population, PED visits slightly dropped from 78 in 2019 to 59 in 2020, though they more than doubled in percentage, from 2.5% to 5.7% (*p* < 0.001) (Table 1). This reduction was mainly related to fewer neonatal pathological conditions, which decreased from 34 (43.6%) to 11 (18.6%), with a complete disappearance of respiratory infections, from 17 to zero. Conversely, visits for minor neonatal clinical conditions increased from 44 (56.4%) to 48 (81.4%), mostly due to feeding-related issues (*p* < 0.001) (Table 2). PED visits of children >14 years were reduced by nearly 83%.

Diagnoses at discharge are detailed in Table 3.

**Figure 2:**
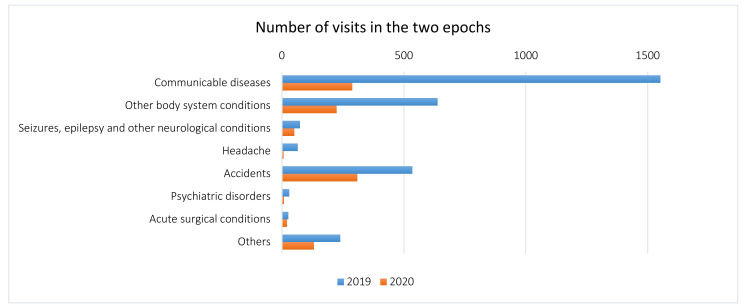
Distribution of diagnoses at discharge in the two study periods (March–April 2019 (non-COVID-19) vs. March–April 2020 (COVID-19)).

Overall, communicable diseases dropped from 1552 (49.1%) to 288 (27.7%), with significantly fewer overall respiratory infections and gastroenteritis (*p* = 0.027 and *p* < 0.001, respectively) (Table 3). Accident-related visits also diminished during COVID-19, from 535 (16.9%) to 309 (29.7%) (*p* < 0.0001), becoming the most common cause of PED visits (Figure 2). In particular, visits for musculoskeletal pain reduced from 113 to 25 (Table 3). Acute surgical conditions slightly diminished, from 26 to 21, even though their relative frequency was increased (*p* < 0.001).

Of note, visits for headaches fell from 65 in the non-COVID-19 period to seven in the COVID-19 period (*p* < 0.003). Other organ system diseases and psychiatric disorders were comparable in the two periods (Table 3).

### 3.3. Outcomes

In terms of outcome, hospital admissions dropped from 266 to 109 during the COVID-19 period, even though there was a slight increase in the hospitalization rate in March–April 2020 compared to March–April 2019 (10.4% vs. 8.4%, respectively: *p* = 0.04) (Table 4). The admission rate in the PICU was comparable in the two epochs (27 (10.15%) in 2019 vs. 11 (10.09%) in 2020; *p* = 0.99). Among the PICU admissions, respiratory issues significantly decreased (13 in 2019 vs. 0 in 2020) (Table 4).

## 4. Discussion

Our study aimed to explore the impact of a strict lockdown policy on patients’ attendance at an urban PED, in one of the first areas of COVID-19 outbreak in Europe. The COVID-19 outbreak in 2020 had a marked impact on the volume and characteristics of visits to our PED in Verona, which dropped to one third of those registered in the same period in 2019. As an unexpected finding, we observed a marked increase in PED visits for minor neonatal clinical conditions, which nearly tripled in percentage compared to 2019. Of note, “accidents” were the most frequent cause for PED visits during COVID-19, exceeding the “communicable diseases” category, which had ranked first in the previous year. The hospitalization rate slightly increased during the COVID-19 period, while admissions to the PICU remained at 10% in both epochs.

After COVID-19 became a pandemic, a drastic decrease in the number of patients presenting to adult EDs was noticed [6]. Similar data were highlighted in the pediatric population [7,8,9]. In our study, we observed a dramatic drop of PED visits by 67% during the COVID-19 period, compared to the same period in 2019. This reduction was undoubtedly linked to the COVID-19 pandemic, as in the two preceding months (January and February 2020) the number of PED visits was equivalent to the previous winter season.

In terms of classification at triage, the white, green, and yellow codes showed a marked reduction in terms of absolute numbers during the COVID-19 period. However, the overall drop in PED visits was mainly due to children presenting with less urgent problems; that is, those classified as white code, which decreased from 45% to 35%, while the percentage of green and yellow codes slightly increased. We may argue that parents of children with minor problems (white code) were more reluctant to take them to the hospital, given the risk of contagion by SARS-CoV-2. Furthermore, accessing hospitals for non-urgent problems was discouraged by restrictions on private and public transport, which limited travel to that required for urgent reasons and essential services only.

Nonetheless, during the COVID-19 period we observed a much larger proportion of otherwise healthy newborns who were brought to our PED for minor neonatal conditions, particularly for feeding-related problems. We believe this somewhat surprising data may be partly explained by the restricted access to primary care services. Additionally, forced home isolation may have limited inexperienced parents’ ability to seek advice from relatives and friends, raising anxiety and worries about the best management for their infant. As a result, need for help around routine care of their baby may have offset parents’ fear of contagion, prompting them to reach out the PED despite the limitations imposed by the lockdown. Yet, by doing so in a COVID-19 context, neonates might have been exposed to higher risk of infection while visiting the PED. Furthermore, inappropriate access to PED for minor neonatal issues could have posed an unnecessary burden to healthcare providers and the hospital system, as well as increased the risk of contagion for other family members. If another lockdown had to be re-instituted, more efficient local services should be made available to limit incongruous neonatal PED accesses.

Unexpectedly, accidents were the most common reason for accessing our PED during the COVID-19 period. Specifically, we found a marked increase in the proportion of injuries, burns, or wounds, suggesting that the home environment remains a frequent place for accidents among children [9,10]. Similarly, other authors observed an increased relative proportion of trauma-related attendances, likely attributed to the lockdown [11,12].

In line with recent findings in large pediatric populations [11,13,14,15], we observed a marked reduction in children presenting with communicable diseases (from 49% to 28%). Avoidance of social interactions, school closures, and suspension of all non-essential services and sport activities may have limited the spread of other viral infections usually seen in the winter season [13,14,15,16].

Nonetheless, communicable diseases ranked as the second most represented category in our PED during the COVID-19 period. In general, infectious diseases are one of the main causes for PED visits throughout the year. Indeed, fever, cough, respiratory distress, vomiting, and diarrhea are commonly observed in children presenting to any PED [16,17,18]. Interestingly, at the pandemic’s onset, the public became quickly aware that respiratory symptoms and fever could be warning signs for COVID-19. Thus, caregivers were prompted to seek PED consultation for children with these symptoms, despite the recommendation to stay at home and the declared lower risk of serious clinical involvement for the pediatric population [19,20,21,22]. Conversely, PED visits for gastroenteritis showed a greater decrease during the COVID-19 period, possibly because parents did not deem gastrointestinal symptoms as possible manifestations of COVID-19, particularly in the first weeks of the outbreak. More information should alert parents and caregivers about the risk of SARS-CoV-2 infection, even in the presence of a typical or specific symptoms, such as vomiting and diarrhea.

Finally, we noticed a significant drop in PED visits for headaches and abdominal and musculoskeletal pain during COVID-19. As these symptoms are frequently related to functional causes in children, reduction in school-related stress may have partly mitigated their occurrence [9,23]. Moreover, despite the strict lockdown policy being associated with adverse psychological effects among children [23,24], parents may not have considered these conditions as severe enough to risk going to the PED during the COVID-19 outbreak. Lastly, fewer musculoskeletal pain presentations may have also been due to lockdown policies allowing no sports.

In terms of outcomes, we observed a moderate increase in the hospitalization rate during the COVID-19 period. This may be consistent with the marked reduction in white codes in 2020, suggesting the population visiting our PED was generally sicker during COVID-19, thus requiring hospital care more frequently. Furthermore, as outlined by other studies [25,26], a lower number of patients required intensive care treatment (27 in 2019 vs. 11 in 2020), even though the PICU admission rate remained unchanged. Of note, the reduction in PICU admissions was related to a striking disappearance of respiratory infections.

This study has several limitations. First, the retrospective study design and the relatively small sample size do not allow us to draw robust conclusions. Second, it is a single center study, thus it may not fully reflect the diversity of care provided in other pediatric emergency departments, in Italy and other countries. Nonetheless, it provides detailed information on the characteristics of over 1000 PED visits in a third-level urban center, during the early phase of a unique situation caused by the COVID-19 pandemic. Our data may be useful for comparing figures from other PEDs, as well as for guidance in adapting healthcare policies for possible future outbreaks. Furthermore, we believe new strategies should be implemented to promote adequate access to health services for children with any pathologic condition, to mitigate the short and long-term adverse effects indirectly connected to fear and unwillingness to visit hospitals due to the risk of COVID-19.

## 5. Conclusions

In conclusion, our study confirmed a substantial reduction in PED visits during the COVID-19 pandemic, but also revealed some distinct characteristics observed in different age groups and disease categories. As a novel finding, we reported an unexpected increase in PED visits for minor neonatal conditions, which highlights the need for primary care services to be more efficient and easily available for caregivers, should other lockdown regimens be established. This would reduce unnecessary PED visits, as well as the inherent risk of contagion for infants and family members. In addition, as one third of PED visits were accident-related, we emphasize the need to implement strategies to raise public awareness of child safety in the domestic environment, particularly during a mandatory stay-at-home policy. Finally, even though the hospitalization rate slightly increased during the COVID-19 period, the PICU admission rate remained unchanged, suggesting an overall judicious use of hospital resources, despite the inevitable pressure PED healthcare providers had to face during the pandemic.

## Figures and Tables

**Table 1:** Demographic characteristics and triage code severity of the pediatric emergency department visits in March–April 2019 (non-COVID-19 period) compared with the same period in 2020 (COVID-19 period). (d = days; y = years).

	2019	2020	*p*-Value
Number of visits, n	3159	1039	
Visits per day, n (SD)	51.62 (10.4)	17.03 (6.8)	<0.001
Sex (n, %)			
Female	1418 (44.9)	437 (42.1)	0.11
Male	1741 (55.1)	602 (57.9)	0.11
Age group (n, %)			
<28 d	78 (2.5)	59 (5.7)	<0.001
28 d–1 y	544 (17.2)	203 (19.5)	0.09
1–3 y	583 (18.5)	241 (23.2)	0.001
3–6 y	860 (27.2)	194 (18.7)	<0.001
6–10 y	436 (13.8)	162 (15.6)	0.15
10–14 y	402 (12.7)	136 (13.1)	0.76
≥14 y	256 (8.1)	44 (4.2)	<0.001
Priority tags at triage			
White (n, %)	1447 (45.8)	364 (35.0)	<0.001
Green (n, %)	1450 (45.9)	574 (55.3)	<0.001
Yellow (n, %)	261 (8.27)	100 (9.6)	0.17
Red (n, %)	1 (0.03)	1 (0.1)	0.41

**Table 2:** Pediatric emergency department visits of the neonatal population (age < 28 days) in March–April 2019 (non-COVID-19) compared with the same period in 2020 (COVID-19).

	2019	2020	*p*-Value
Total visits, n	78	59	
Newborns with pathological conditions, n (%)	34 (43.6)	11 (18.6)	<0.001
Respiratory infections	17 (50)	0 (0)	0.016
Urinary infections	2 (5.9)	0 (0)	0.71
Aspecific fever	3 (8.8)	1 (9.1)	0.99
Other infections	7 (20.6)	3 (27.3)	0.64
Neurological conditions	2 (5.9)	3 (27.3)	0.08
Others	3 (8.8)	4 (36.4)	0.03
Newborns with minor clinical conditions, n (%)	44 (56.4)	48 (81.4)	<0.001
Aspecific crying	3 (6.8)	0 (0)	0.26
Umbilical cord care	13 (29.5)	9 (18.8)	0.22
Feeding issues	10 (22.7)	25 (52.1)	0.007
Constipation	12 (27.3)	7 (14.6)	0.13
Others	6 (13.6)	7 (14.6)	0.9

**Table 3:** Pediatric emergency department diagnosis at discharge in March–April 2019 (non-COVID-19) compared with the same period in 2020 (COVID-19).

Diagnosis at Discharge, n (%)	2019	2020	*p*
Communicable diseases	1552 (49.1)	288 (27.7)	<0.001
Lower airways infections	255 (16.4)	54 (18.7)	0.33
Upper airways infections	796 (51.3)	160 (55.6)	0.18
Gastroenteritis	378 (24.4)	42 (14.6)	<0.001
Aspecific fever	123 (7.9)	32 (11.1)	0.07
Other body system conditions	638 (20.2)	224 (21.6)	0.34
Gastrointestinal and genitourinary diseases	371 (58.2)	159 (70.99)	0.01
Dermatological pathologies	247 (41.8)	65 (29.01)	0.01
Seizures, epilepsy and other neurological conditions	74 (2.3)	51 (4.9)	<0.001
Headache	65 (2.1)	7 (0.7)	0.003
Accidents	535 (16.9)	309 (29.7)	<0.001
Head injury	132 (24.7)	71 (22.98)	0.58
Other traumas—burns—wounds	237 (44.3)	183 (59.22)	<0.001
Foreign body ingestion/inhalation	53 (9.9)	30 (9.7)	0.92
Musculoskeletal pain	113 (21.1)	25 (8.1)	<0.001
Psychiatric disorders	30 (0.9)	8 (0.8)	0.59
Acute surgical conditions	26 (0.8)	21 (2.0)	0.001
Others	239 (7.6)	131 (12.6)	<0.001
Total	3159	1039	

**Table 4:** Outcome of the population accessing the Verona pediatric emergency department in March–April 2019 (non-COVID-19) compared with the same period in 2020 (COVID-19).

Outcome, n (%)	2019	2020	*p*
Discharge	2893 (91.6)	930 (89.5)	**0.04**
Admissions	266 (8.4)	109 (10.5)	**0.04**
PICU admissions	27 (10.2)	11 (10.1)	0.99
Respiratory conditions	13 (48.1)	0 (0)	**0.02**
Prolonged seizures	1 (3.7)	2 (18.2)	0.13
Sepsis	3 (11.1)	2 (18.2)	0.29
Major trauma	2 (7.4)	3 (27.3)	0.1
Diabetic ketoacidosis	0 (0)	1 (9.1)	0.5
Surgical urgencies	4 (14.8)	0 (0)	0.37
Others	4 (14.8)	3 (27.3)	

## Data Availability

The datasets used and analyzed during the current study are available from the corresponding author on reasonable request.
